# Valedictory Address of the President of the Illinois State Medical Society

**Published:** 1860-08

**Authors:** David Prince

**Affiliations:** Jacksonville. Ill.


THE CHICAGO
MEDICAL EXAMINER

VOL. I. AUGUST, 1860. No. 8

### ORIGINAL COMMUNICATIONS.

---

### REMARKS UPON SOME OF THE LEGAL RESPONSIBILITIES OF MEDICAL MEN.—Being the Valedictory Address of the retiring President of the Illinois State Medical Society, read at the Annual Meeting in Paris, May 1860.

---

By DAVID PRINCE, M. D., of Jacksonville. Ill.

---

The medical and surgical practitioner of generous and honorable feelings, is apt to think that his patients will feel towards him as grateful for attention to them in times of distress, as he in turn would feel if in like trouble, and attended with equal assiduity. Perhaps it is not claiming too much for human nature to say, that this expectation is not unfounded and not disappointed with regard to the better portion, and it may be the larger portion of patients. The physician often meets with expressions of gratitude for services rendered, where he sees on reviewing the case that he ought to have treated it much better, and he feels a little pricked that he should be so unworthy of the expressions of gratitude and confidence.

Estimating the world by the better portion of it, and stimulated by testimonials of appreciation for services rendered to the grateful and to the just, he is liable to be thrown off his guard, and to fail to surround himself with those provisions and proofs which he would never neglect if he stopped in every case to reflect that it might become the subject of legal investigation. His education fails to impress him with the importance of being able to prove what he says and does, and the necessary habits of his daily professional life still further confirm this want of caution. Many of his most important consultations with his patients are strictly private, and it becomes a repulsive thought to him, that when he goes to relieve distress, and remove or prevent deformity, or save life, he must take along with him outside witnesses by whom he can prove what promises or proposals he makes, and what measures he resorts to. It is hard, indeed it is nearly impossible for him to adopt the commercial maxim, to treat every man as a rogue until he proves himself honest; to regard every patient as an ingrate or a villain, until he shows by his honorable conduct that he is endowed with honorable and generous feelings.

The doctor is under the further temptation to hold out to the patient, and the immediate friends by whom he is surrounded, the best view of the case. He well knows the depressing effect of an unfavorable prognosis gloomily expressed, and it requires a cold calculation to make the case as bad as it is, or worse than it is, in presence of the patient; for the professional and legal advantages which may afterwards result to him, though the chances of these advantages may lessen the patient's chances for recovery. Many a man will die if his physician thinks so, who will at the same time recover if his doctor thinks so, or if he tell him so.

The physician is led on by these habits and these considerations, thinking, saying, and doing the best things for his patients, never taking caution to guard or to prove his words or his deeds; and he is some day surprised to find himself overwhelmed with a slander, and he looks about him for his proofs wherewith to refute the falsehoods industriously circulated against him, and to his astonishment he finds that he has no proofs but his general character. He can only set his general conduct against the particular lie. He pays little attention to this, thinking it easier to live down a lie than to attempt its formal refutation, especially as he has been utterly careless to guard himself by testimony during the progress of the particular transaction. He learns no lesson of caution from this exhibition of malice. He lives and works, and is believed and appreciated, while the lie is forgotten, and the slanderer is despised: but he is some day brought to a stand by a prosecution, which, if successful, will sweep away his hard earnings through a course of years, and rob him of his well-earned reputation, which to him is the means of future employment and livelihood. He looks about him for his witnesses, and he finds that out of pure goodness he promised too much in the case, and failed to tell all he knew of the dangers and difficulties, in order that the patient might have the greatest solace during the period of confinement and suffering. He finds that the facts in his favor can only be proved by his own assertion and that of the prosecuting parties, so that in law they might as well not be facts. He ascertains that the facts which he can prove are of such a nature as not readily to be appreciated by the members of a jury not especially instructed in the art, and he fears that out of jealousy a neighboring practitioner will take advantage of his privileges before the jury to injure the reputation of his unfortunate rival.

He finds that the public expect perfection of result, unless the practitioner has distinctly stated the difficulties which make this uncertain or impossible, and that the legal tribunal will hold him to this strict rule, unless he can prove by competent witnesses that he has guarded himself, or by the testimony of experts that this perfection of result is under the circumstances impossible.

Where this perfection of result is not secured, he finds himself, in many instances, held socially and professionally responsible, unless his prudence has been such as to be offensive to the feelings of honorable patients, and on the verge of impairing his efficiency and usefulness as a practitioner.

In this country a civil or a criminal prosecution based upon alleged wrong-practice in medicine, surgery, obstetrics; in the practice of the apothecary, and in all other arts, sciences, and trades, is carried on before a jury, none of whom are required to know by preliminary education or training, anything about the matter which is brought before them.

The question whether one telegraphic machine is an infringement upon the patent right of another, may happen to be tried before a jury of farmers and others, not one of whom may have been instructed in mechanics and electricity. A question whether an operation in surgery has been properly performed under proper circumstances, is generally tried before men who are ignorant of the rudiments of anatomy and of all other branches of medical science. This system looks absurd enough, and would leave the dicision almost entirely to chance, or to the trickery of the lawyers, but for the relief found in the admission of testimony by "experts." In any question before a jury, involving any art or science not supposed to be familiar to ordinary men, the parties to the suit are permitted to call before the jury persons who are supposed to be familiar with the principles and rules of the particular art, science, or trade in hand, and to express their opinion in answer to hypothetical questions. The expert upon the witness stand, is sometimes permitted to read from books upon the particular art or science in support or explanation of his own views, but according to Elwell, in his recent work on malpractice, there is no uniform rule with regard to this in our courts. This testimony by reading from books is as often rejected as admitted. (Elwell, p. 331.) Were a jury of men uneducated in the matter in hand, capable, during the few hours that they listen to explanations by experts, of getting clear ideas of the art or science attempted to be explained, the remedy would be nearly perfect. It may have taken years for the expert to acquire the knowledge upon which he bases the opinions he gives to the jury, and he may find it necessary to his own truthfulness and reputation to guard his opinions with many provisos.

These ideas and opinions, many of which have no common words by which they can be expressed, may fall upon the ears of a jury like a talk in an unknown tongue.

That the verdicts of juries thus constituted, though instructed by the testimony of experts, is extremely uncertain, is proved by daily observation. In illustration, I quote in substance two cases from Elwell's work on malpractice.

Pages 81–82. A man had his leg crushed by a log rolling over it. The injury was so severe that the surgeons amputated the limb to save the man's life.

Some years afterwards the bones were dug up and made the basis of a prosecution for the recovery of ten thousand dollars damages.

Emiment counsel were found to undertake the case for a portion of the spoils. Several long trials were had, the juries not agreeing. Depositions were taken in New York, Philadelphia, and Washington, involving great expense. No judgment was obtained against the defendants, but the litigation was ruinous to them. Had these surgeons made the hazardous attempt to save the limb, and had they succeeded to a tolerable degree, then they would have been sued for not performing a perfect cure.

In another case a young man fell with his leg beneath a colt, resulting in a compound cominuted fracture of the tibia fibula, rupturing an artery, and so injuring the leg that the foot became cold. The surgeon made the hazardous but successful attempt to save the limb. Extensive suppuration and sloughing ensued. Extension was out of the question.

The patient recovered at length, through the most assidu ous attention on the part of his surgeon, with a limb half an inch shorter than the other, and a "healing" ulcer over the instep.

The surgeon received from the authorities twenty dollars for his services, the patient being a pauper. So soon as the patient could travel, he found his way to a lawyer and commenced suit against the surgeon, not because he had not cut off the limb, which he should have done according to the best rules of surgery, but because the limb was half an inch too short, and there was an ulcer still remaining. Damages claimed, $5000. The case after hanging several terms was finally dropped, and the surgeon, disgusted, retired from the profession.

Taking the law as it is, and the courts and juries as they are, what is the course the practitioner should pursue in justice to himself and to the public? I ask in justice to the public, because it is the highest interest of the public, that the votaries of any art or science should be shielded from exposure to ruinous or unjust prosecutions—ruinous in expenses to the defendant if the prosecution is unsuccessful, and utterly ruinous in reputation as well as money, if successful.

I am happy to be able to read an extract from a letter recently received from Dr. R. D. Mussey, for fifty years a professor of surgery, recently of Cincinnati, and now of Boston:

"Several years before I gave up teaching medical classes, I urgently desired them, when called in cases of fracture, either to decline taking charge of the case, or to state to the patient and friends before a number of competent witnesses, that they could not promise a satisfactory result; but if their services were desired, they would do the best they could: or, if during the treatment, the directions were so far neglected as to expose the case to a bad result, to go to the patient attended with *reliable witnesses*, and dismiss themselves from the case; of course assigning the reasons. I am now out of all this matter, but if I were not, I would invariably pursue this course."

Prudence, doubtless, equally demands that in all cases in which subsequent legal proceedings may be supposed possible, especially before the resort to important surgical operations, and before a resort to measures which are new or not well settled in the profession, and before a resort to the extreme measures of turning or delivery by instruments in obstetrics; the practitioner should guard him as carefully as Prof. Mussey advises in cases of fractures. It is of course unnecessary to do this with formality or ostentation.

If the necessary witnesses are accidentally present, it may be better to use them than to call others; and if not present, they should be sent for as assistants, or for the moral support of their presence, rather than ostensibly as witnesses. The remarks which the practitioner may think it necessary to make, in order to guard himself against future legal proceedings, ought often, if not generally, to be made out of the hearing of the patient, except in cases of injuries. But to call the father or husband aside and talk privately to him will not answer the legal purpose. Some disinterested friend must hear the consultation.

In case there is a consultation of physicians, and the result is stated to the patient or legal practitioners, and the measures recommended are assented to, this assent, thus capable of being proved, puts the case beyond successful prosecution.

A case may be supposed to be of doubtful diagnosis, and still more doubtful prognosis, and a surgical operation may be deemed necessary to save the patient from death which may be considered otherwise certain.

If all the doubts and difficulties are explained before competent witnesses, and the patient or his proper guardian takes the judgment and skill of the practitioner, as is confessed to be imperfect, for the best benefit he can get from them, and not as perfect and unerring, the doctor is safe from successful prosecution, however erroneous his judgment or practice may be proved to have been. It may in some cases be wise and prudent to require a pledge before hand, that there shall not be even *social or professional blame*, if the case should turn out unfortunately.

If the mouths of the patients and friends are thus sealed in advance, many slanders will be obviated, and many vexatious prosecutions will be avoided; and when they are not avoided, the practitioner can enter upon the defence with confidence, knowing that he can prove his points.

We have to take human nature, the law, the courts, and the juries as they are, and shield ourselves from their abuses the best way we can.

But we are in an age of change and progress, and it is not too much to hope for a change in our jurisprudence. The change which we need is pointed out in the following extract of a letter written by Dr. Alfred S. Tayler, (author of a work on Medical Jurisprudence,) to Sir James Clarke, at the instance of Dr. A. McFarland, now of the Illinois Hospital for Insane, dated London, April 18th, 1854.

"There are three classes of cases which come before our tribunals, Surgical, Medical and Obstetrical, but the first and last are by far the most common, as evidence with regard to proper or improper treatment is in them reducible to greater certainty.

Medical men are often most unfairly dealt with on these occasions, and the unfairness proceeds from three sources. 1st, the Judges, 2d, the Juries, and 3d, members of their own profession appearing as witnesses against them.

1st. The Judges in many cases take a severe view of the matter in action; and so far as I know they invariably rule in favor of the quack, and adversly to the educated practictioner. The ignorance and want of education in a quack, are actually assigned as reasons for treating him leniently! A regular practitioner charged with *mala praxis*, if a verdict be returned against him, is heavily paid or punished, because, as the judge tells him, his professional education should have taught him! A mistake on his part was the more culpable, because he had had the opportunity of acquiring knowledge, which was denied to the quack. So far as I know, the law is uniformly laid down in this manner by the judges.

If a quack and a surgeon be tried for the same kind of *mala praxis*, involving the same civil injury, or the same criminal responsibility, the judges, in passing sentence, would advise the quack not to take up the practice of an art which he did not understand, at the same time, as he had good motives, and knew no better, his offence would be leniently dealt with.

The unlucky Surgeon would be told that his ignorance was of the most culpable kind, that in proportion to the opportunities he had of acquiring information by a regular surgical education, the greater was the disgrace and infamy of his conduct, and a severe punishment or heavy damage would be inflicted upon him.

The judges and the law are then decidedly against the regular practitioner.

2d. The *Juries*. Whether the jury be common or special, as it is ordinarily constituted, its members are not competent to pronounce upon the propriety or impropriety of certain methods of treatment. They cannot possibly enter into the professional reasons, which induce medical men to adopt a line of treatment that may be objected to as *mala praxis*, and yet, under our system of jurisprudence, they are left to grope their way to a verdict, in the dark. The damages which they assign are generally heavy and disproportionate.

* * * The provoking part of the case is that there are other and inferior professions in which a totally different system is pursued. In any collision at sea or on the river between two vessels, the captain of one being charged with unskillfulness in navigating his craft, and damages being sought against him—a common or special jury at *nisi prius*, is not allowed to decide such a case. They are not permitted to determine whether the helm should have been port or starboard, or whether the fore sail or the jib was wrongly set, and thus led to a collision.

On the contrary, Masters of the Trinity House are called to the assistance of the admiralty judge. They sit as assessors and inform the judge of what the proper course of navigation would require in such cases.

3d. *Medical Witnesses*. They are often bitter enemies to members of their own profession, and lawyers delight in playing the doctors off against each other. Each solicitor can select his own witnesses, a bad case is often hawked about the profession for some days, but it will pretty surely find one or more supporters in the end, owing to strong exparte statements being laid before the medical men consulted.

My opinion is that we shall have no reform of this bad system, until a Board of Medical Assessors, (consisting of men of high repute in their respective departments,) is appointed. This would abolish the three classes of evils which create so much ground of complaint."

The defects in our jurisprudence, with regard to jury trials, may be to some extent remedied in several obvious ways.

I will suggest but two of them :

1. In any case the correct verdict upon which involves a greater familiarity with the art or science in question than is common to men of ordinary general education to require as a qualification of a member of the jury, that he have had a special training or education in the particular art or science. This would diminish the necessity for testimony by experts, which it is often difficult to procure in person, while it is necessarily imperfect when obtained by deposition. With such a jury the case would be far less likely to turn upon the mere shrewdness or power of ridicule of the lawyer.

2. Another method would be, to so constitute our higher courts, or a branch of them, that the parties may appeal from the lower courts, in order that questions of right or wrong practice in any particular art or science may be passed upon by a jury or board of experts, free from the prejudices incident to the immediate neighborhood of the transaction.

This latter investigation may be based upon the recorded testimony with regard to facts taken in the lower court, and need not necessarily involve ruinous expense.

The defects and abuses of our jurisprudence bear no harder upon medical men than upon all others whose occupations lead them to practice arts unfamiliar to ordinary men.

But from the peculiar relations of the medical art to life and health, while its principles are so entirely hidden to those uneducated in it, the temptations to prosecutions against practitioners of the healing art are very great.

Next to these are the captains, pilots, and engineers upon our lakes and rivers, and the conductors, engineers, switchmen, &c., upon our railroads.

The rules and principles concerned in these arts are comparatively few and simple, and yet common juries often fail to get a clear understanding of them from the best explanations which experts can give them from the witness stand.

If it is difficult for the members of a jury to acquire a knowledge of these during the few hours a trial may be in progress, how impossible it must be for them to become acquainted with chemistry, anatomy, pathology, therapeutics, surgery, and Obstetrics! And yet, without clear ideas of some or all of these subjects, their reasoning and their verdict are alike all in the dark.

It is notorious that the result turns more upon the ability and skill with which a case is managed, than upon the inherent justice of the case.

It is the interest of all classes to have this bungling and uncertain system abolished, or materially amended.

While the medical man would be saved from much needless injustice, the public would also be saved from much groundless or selfish expectation of unearned and undeserved awards of "damages," where no damage has been received, and from much misdirected labor and anxiety.

Were our jurisprudence so ordered that justice would be the rule, and a wrong verdict the exception, none but aggravated cases of ignorance or carelessness would usually be prosecuted, and the sufferer thinking himself aggrieved, would first take counsel of an adviser versed in the science or art in question, rather than of a sharp-nosed advocate, who would be ready to undertake the case for a share of the profits.

